# Health Complex Model as the Start of a New Primary Healthcare Reform in Iran: Part B: The Intervention Protocol

**Published:** 2019-01

**Authors:** Jafar Sadegh TABRIZI, Majid KARAMOUZ, Homayoun SADEGHI-BAZARGANI, Alireza NIKNIAZ, Leila NIKNIAZ, Roya HASANZADEH, Jalal HANAEE, Mostafa FARAHBAKHSH

**Affiliations:** 1. Tabriz Health Services Management Research Center, Health Management and Safety Promotion Research Institute, Tabriz University of Medical Sciences, Tabriz, Iran; 2. East Azerbaijan Province Health Center, Tabriz University of Medical Sciences, Tabriz, Iran; 3. Road Traffic Injury Research Center, Health Management and Safety Promotion Research Institute, Tabriz University of Medical Sciences, Tabriz, Iran; 4. Department of Pediatrics, Faculty of Medicine, Tabriz University of Medical Sciences, Tabriz, Iran; 5. Department of Medicinal Chemistry, Faculty of Pharmacy, Tabriz University of Medical Sciences, Tabriz, Iran; 6. Research Center of Psychiatry and Behavioral Sciences, Aging Research Institute, Tabriz University of Medical Sciences, Tabriz, Iran

**Keywords:** Health complex model, Primary health care, Reform, Iran

## Abstract

**Background:**

For overcoming the existing problems and finding a pathway for realization of universal health care, health complexes were implemented in the form of a pilot project in Tabriz suburban area.

**Methods:**

Tabriz Health Complex Project was designed in 2013 in the provincial health center of East Azerbaijan. In terms of execution schedule, this intervention had 4 phases including 1) study phase, 2) planning phase, 3) pilot phase, and 4) implementation phase. Each health complex covers a population of 40,000 to 120,000 in a defined geographic area and consists of a Comprehensive Health Center (CHC) including health centers and a management center, which usually located in CHC. The important features of this project are as follows: people-centered primary health care, special attention to health promotion and prevention and establishment of a referral system within the region (organic connection between the first and second levels).

**Results:**

An accountable and responsive health care system has been established to deliver integrated care services to people in a defined catchment area against identified per capita payment, under district health centre policies and regulations. Each health team consisted of a general practitioner and a family health nurse who covered around 4000 people to deliver prevention, promotion, and treatment services especially in and NCDs field.

**Conclusion:**

Health complex as a model of public-private participation and practical solution to address many of the problems in the primary care system of the country. The project can organize the PHC system and family medicine program.

## Introduction

With the adoption of the Alma-Ata Declaration in 1978 and the commitment of the member states of the WHO to achieve “health for all by the year 2000”, an international movement was started to achieve health with Primary Health Care (PHC) (1). By implementation of PHC in rural areas, Iran was one of the leading countries in the world. With all progress in rural areas, little success was achieved in providing primary health care in urban regions and services in the second and third levels faced with numerous challenges and difficulties (2,3).

Family Medicine is considered by the WHO as the center of global efforts to improve the quality, effectiveness, equity and reduce the cost of health care systems (4). Therefore, in order to solve the problems of the referral system, develop PHC in rural areas, implement active services in urban areas and ultimately, greater public benefits, family physician program was adopted by Iran’s Islamic Consultative Assembly and implemented in rural and urban areas with a population less than 20000 residents since 2005. Based on numerous studies on family physician programs in rural and urban areas, it seems that after 8 years of implementation in rural areas and 2 years in urban areas in the provinces of Fars and Mazandaran, the program has faced numerous challenges and there are considerable differences with what should have been implemented (5).

On the other hand, Public-Private Partnership (PPP) in the provision of health care, is the issue of the day in many countries and new and creative thinking in health promotion (6–9). PPP is a combination of public and private organizations created to finance and provide health services to increase efficiency and more health (10). One example of successful PPP in the health system is health care cooperatives were designed at the Tabriz University of Medical Sciences. They were created in 1998 with cooperation of the Ministry of Cooperatives in order to create a good relationship with the private sector and encourage them to move in line with the government's macroeconomic policies to improve health (6). Considering the general policies in the health system transition map in terms of leveling of services, family physician and referral system, changes in the structure, attitudes, and policies towards the use of resources seems inevitable (11). Hence, Tabriz University of Medical Sciences framed health complexes for overcoming the existing problems and finding a pathway for realization of UHC and it was implemented in the form of a pilot project in Tabriz suburban area.

## Methods

This protocol study describe the establishment of new primary health care model in Iran. For the design of Health Complexes (HC), previous experiences of Tabriz University of Medical Sciences in commissioning health care cooperatives, national experiences in family physician program and experiences of numerous countries including the United Kingdom, Canada, Australia, Turkey, Netherland, Sweden, etc. were reviewed and positive aspects of each were extracted. Eventually, the health complex plan was developed with the ultimate goal of universal health coverage promotion, public health promotion and increasing the satisfaction of recipients and providers of health services.

### Objectives

Tabriz Health Complex Project (THCP) was designed in 2013 in the provincial health center of East Azerbaijan and it was implemented with the participation of other departments of Tabriz University of Medical Sciences like: Education affair, Logistic affair, Treatment affair and organization of Iranian health insurance and private sector.

Specific objectives were as follows: Expanding services coverage, expanding population coverage, expanding financial protection, reforming the structure and procedures to benefit from the resources, modification of the payment methods, improving behavior of health care providers and patients, improving quality of services.

Therefore, Tabriz University of Medical Sciences as a custodian of health services at the provincial level could create a major change in providing services, accountability to the people within the framework of family physician program, and referral system with a bold mindset change from “governmental service delivery” to “ensuring standard service delivery”. Phase b is a prospective follow-up study in which data recollection will be performed every 2 years after the intervention initiated.

### Intervention Time bound

In terms of execution schedule, this intervention had 4 phases including 1) study phase, 2) planning phase, 3) pilot phase, 4) implementation phase (Table 1).

**Table 1:** Different phases of intervention and main activities to each phase

Intervention Phases	Related activities
Study	Formation of “provincial family physician committee” and it’s 9 specialized subcommittees
	Assess of good experiences of family physician in the world
	Assess of Iran’s experiences in PHC and family physician
	Examine the existing situation in the intervention areas
	Designing an urban family physician model based on available evidence and opinions of experts in the country
Planning	Calculate per capita payment developing 5-year plan
	Developing implementation guidelines for the 5-year project
	Developing project action plans
Pilot	Public health complexes in Osku city and Rajaee city district in Tabriz
	Health complex with the public and private partnership in the new Sahand city and Akhmaghie and Ravasan district
	Monitoring and evaluation of program during and after the implementation of pilot programs in the designated areas
Implementation	Widespread implementation of the program after eliminating weaknesses and enhance strengths derived from the pilot phase

In the study phase, after the formation of “provincial family physician committee” with directing of the head of Tabriz University of Medical Sciences, 9 specialized committees were organized.

These committees were as follows: Policy Committee, Structure Committee, Referral Committee, Guideline Committee, Education Committee, Health Economics, and Resources Committee, Information technology Committee, Monitoring and Evaluation Committee, Culture-building and Notification Committee.

One of the strengths of this project was the application of all capacities and chancellors of the university and engaging different affairs of university in all policy committees. In the following phase, international experiences and good models of family physician programs in the world were evaluated by a comparative study. Iran’s experiences regarding primary health care and family physician program were assessed and then researches were being done to assess the situation of intervention areas. Finally, at the end of study phase, a model for the implementation of urban family physician was designed based on evidence and opinions of experts and specialists in the country.

In the planning phase, after calculating the per capita payment by using scientific and modern methods and according to the country's upstream documents, 5-year plan for the project was written. Then, its implementation guidelines and action plans were developed to be applied in a pilot phase.

The pilot phase of the program was in 2 forms: 1) fully governmental health complex in the Osku city and Rajaee Shahr in Tabriz and 2) Health complex with the public and private partnership (PPP) in the new Sahand city (located in the Osku city) and Akhmaghie and Ravasan district (in outskirt of Tabriz). To assess the effectiveness of the intervention, control area was also considered in the baseline and final evaluation.

Azarshahr for the fully governmental health complex and for the private residence of the Imamieh district in Tabriz for public and private partnership were considered as the controls Monitoring and evaluation of program will be carried out during and after the implementation of pilot programs in designated areas according to specific instructions (self-evaluation, internal and external evaluation). After the elimination of weaknesses and enhancing strengths, the program will be implemented widely (Table 1).

The project was implemented in Jun 2014 in Akhmaghie and Ravasan area located in the southwestern margin of Tabriz, Iran. This project represents a comprehensive Public Private Partnership (PPP) in regional health management, planning, service delivery, training, research, and evaluation.

### Ethics approval

The study protocol was approved by Ethics Committee of Tabriz University of Medical Sciences (1394.383). All subjects will make aware of the content of the study and a written informed consent document will be obtained. The results of this survey will be presented as research articles and reports for policymakers.

## Results

In this new structure called Health Complex (HC), an accountable and responsive health care system established to deliver integrated care services to people in a defined catchment area against identified per capita payment, under district health center policies and regulations.

The features of this project are as follows: the position of public participation and organizations involved in health in creating a healthy environment for the promotion of public health in the region, people-centered primary health care, special attention to health promotion and prevention, establishment of a referral system within the region (organic connection between the first and second levels), creating electronic health records for all covered individuals, establishment of level by level, coherent and purposeful evaluation system and performance-based payment system.

### Organizing and defined population

Each HC covers a population of 40,000 to 120,000 in a defined geographic area and includes a Comprehensive Health Center (CHC) health centers and a management center, which usually located in CHC.

The management center is actually the first level of management where health experts (family and school health, diseases, occupational and environmental, logistic and financial support experts) are located in. Based on population density, 2 to 5 health teams (8,000 to 16,000 people) are classified in one health center. Each health team consisted of a general practitioner and a family health nurse who covered around 4000 people to deliver prevention, promotion, and treatment and NCDs care services (Table 2).

**Table 2:** Presented care package in Health Complex

***Service package***	
Self-care services	Urgency services in disasters
Targeted screening and identification of risk factors	Consultation and care of diet and physical activity
Recognizing and treatment of acute and chronic disease	Consultation and care of social health
Pregnancy and childcare	Maintain and improve the management of infectious diseases
Middle-aged and elderly care	
Consultation and mental health care	Rehabilitation services
Occupational and Environmental Health Services	Outpatient and minor surgery procedures
Referral and Specialized outpatient treatment	Oral and dental Health Services
Pharmaceutical and Paraclinical care	Home care services
Emergency services	

One of the physicians is in charge of the center. The defined health center in the defined geographical area offers the defined service packages to the population covered under the management of health complex committee.

CHC is one of the health centers that are suitable and accessible for delivering integrated care services. 24-hour emergency and ambulatory services and referral specialist clinic with at least four specialists (internist, pediatrician, gynecologist, and psychiatrist) are located in this center. These specialists visit patients referred from health centers according to specified weekly schedule and send the relevant feedback to the referral physician. In addition to accepting referrals, specialists are responsible for monitoring family physician referrals, follow-up of referred patients, training of family physicians and responding to their telephone counseling.

**Fig.1:**
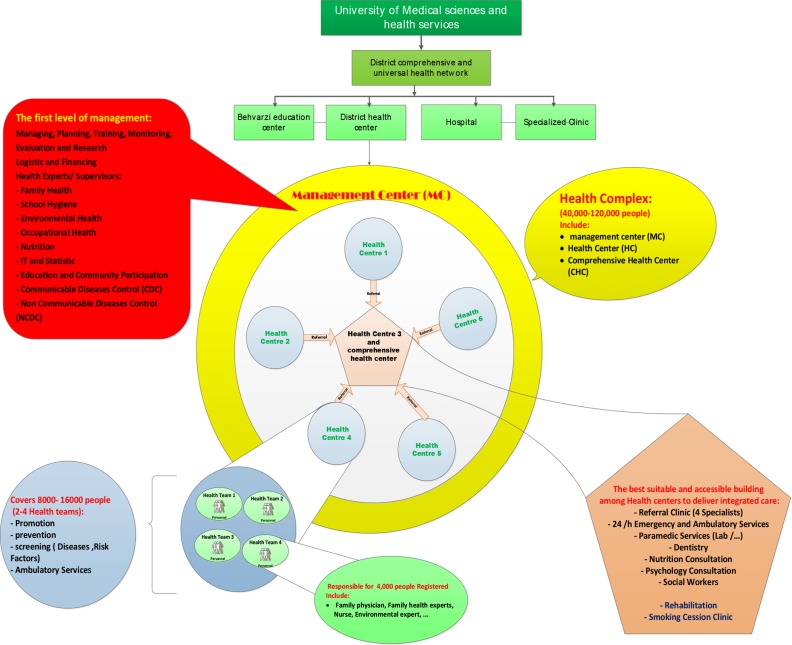
The health complex structure (20)

Moreover, in each CHC, a dietitian, a mental health professional, and an occupational and environmental expert have covered the services. Nutrition and psychiatric consultation, dentistry services (free of charge identified services for children under 14 yr old and pregnant women) and some social services are delivered in CHC as well (Fig.1). Health complexes can be administered as public, private, cooperative or a combination of the above. Table 3 represents the minimum expected staff for health complexes; however, health complexes can use additional staff in different categories with different expertise according to their needs and higher accountability.

**Table 3:** Expected workforces from Health Complexes

***Profession***	***Health center***	***Management center/CHC***
General practitioner/Family physician	1 for 4,000 people	--
Midwife/Family health technician	1 for 4,000 people	--
Occupational and environmental health technician	1	2
Health service manager	--	1
Diet consultant	--	2
Mental Health	--	2
Nurse	1	--
Specialist	--	At least 4

### Training of workforce

Training programs in the beginning and during the defined services based on the service packages is anticipated. All the staff should pass the 6-day course before employment for obtaining the related certificates. The training during service scheduled in two parts including an 18-day period of getting familiar with the service packages two times a week. The second part of training is continuous based on the persistent education programs.

### Payment method

In order to cover and reduce disadvantages of various methods and to achieve the desired financial objectives, mixed payment method including per capita, fee for services and bonus payments have been used in this project (12). Capitation method is used for health promotion, prevention, risk factors and diseases screening, and specialists’ services. Bonus payments is applied for special activities related to the regional health problems and needs and district-specific programs. As well, fee for services is ambulatory services, medication, paramedic services and rehabilitation.

Per capita and bonus payments are paid based on the city health center contract and fee for services are paid based on contracts with insurance organizations. Nearly 80% of the per capita payment is paid to health complexes monthly and the remaining 20% is paid based on conducted evaluations (quantity and quality of services).

Clients pay a part of the service cost to service providers in the form of franchise while receiving the service. In outpatient care, it is 10% of the state tariff and in para clinic, it is about 30% of the state tariff. Method of payment to health complex staff is in the form of salary, per capita, fee for services, reward and payment based on performance.

### Monitoring and evaluation

Evaluation is based on the service packages. Monitoring and evaluation are performed through 2 ways: Public oversight and experts’ evaluation. Public oversight is based on the services expected from the health complexes provided to all families in the form of booklets. The booklet included service type, address for centers, main features of services, customer service flowchart and customer rights.

Specialized monitoring and evaluation are based on the service packages and the expected standards in the evaluation checklist performed at three steps. The first step includes self-assessment in which staff will assess their services monthly. The second step is the internal evaluation where the management of the center evaluates the health centers every two months. Finally, the third step is the external evaluation performed by experts from district health center in collaboration with the provincial health center every three months. The remaining 30% of per capita will be paid to the health complexes’ based on the results of external evaluation. Internal evaluation is done by observers in the health complex. In each complex, there are 3–4 supervisors who received training on the monitoring and service processes. External evaluation is carried out by the supervisor of the Tabriz health center.

### Financing

In order to better manage the financial resources, avoidance of multiplicity in financial decisions and standardization of payments, all credits of insurance organizations and the Ministry of Health will be pooled in the same fund and administered by provincial policy and executive committee.

## Discussion

Tabriz Health Complex Project with a unique structure is a strategy to achieve Universal Health Coverage (UHC) and establishment of reformed family physician program (13).

The highlight features of THCP that contributed in improvement of the health care system and solving the major problems of the current PHC system (Increased out of pocket payments, lack of providing comprehensive services, false policy making and etc.) are as follows:

1. Baseline evaluations with taking account a control group is one important feature of health complexes. The community-based evaluation was used for assessing health complexes. In other words, all people should inform about provided services by health complex in order to ask for the required services. The content of monitoring and evaluation are as follows: client satisfaction, employee satisfaction, quantity and quality of services at all levels, medication, paraclinical, compliance with clinical guidelines, and referrals. Improving public-private partnership and participation of the private sector in the provision of primary health care (10, 14). Actually, changing the direction of university from service delivery to ensure optimal service delivery. In India, one of the important reform strategies was collaborating with the private sector in the form of PPP. In the health sector, the PPP, as a social entity, pools the best features of the two merging authorities of government and private sectors already shown their potential for accountability (15).
2. Suitable, transparent and coordinated communication between different levels of services by establishing appropriate and responsive referral system; therefore, participation and commitment of three levels of services are provided to improve quality and deliver all society needed services.
3. Providing a comprehensive package of services at different levels (education, research and services) as well as development of the service packages (including health promotion, prevention, treatment and rehabilitation) to all defined population with concentrated financial support. Therefore, governments have been asked to move toward increasing the coverage of services, increasing coverage of people and reducing out of pocket payments (13).
4. Patient detection and basic visits are important aspects of this intervention. According to determined service packages, responsibility and accountability are also increasing.
5. Developing a comprehensive electronic health file, health record and report system are the other characteristics of THCP. Electronic health records and reporting systems used in many developed countries (16, 17), leads to many benefits, including fast, convenient and effective referrals, safer clinical data transmission, not missing referral papers during transfer, receiving a standard set of data on each patient, easy access to general practitioners, logistic and support system for general practitioners and managers, improving the quality of referred information because of more readable and more completed information, integrated system with existing family practice systems and improving patient safety and quality of care.
6. Establishing a systemic thinking and integrated stewardship in health system are other important aspects of health system reform. In the other words, administrative roles of health system such as supervision, monitoring, evaluation, delegation, coordination and etc. could be transferred from district to regional authorities and service purchasing from public/private sector.
7. The reform of provider payment methods (mixed payment method) and establishing a pay for performance system (pay for care of each condition in the right way, not necessarily the same way for all conditions (18), and strategic purchasing of services are other important features. With strategic purchasing of basic health services, limited health resources are used to provide maximum efficiency in the health system. Fairness in distribution of health care is possible, resources are targeted, quality of health services is improved, payment methods be reformed, demand (especially in the field of medicine and laboratory services) is managed, and induced demand and out of pocket payments are reduced (17).
8. Social Emergency Referral System (SERS) and control of social factors are provided in this system. Nowadays, more than 9% of the total population of East-Azerbaijan Province are in the elderly age group. They have specific health care needs and require planning and implementation of specific health care programs. In THCP, some health centers are selected as “Elderly friend centers”. These centers work in the field of prevention, diagnosis and treatment of diseases of the elderly, with the aim of increasing the social living conditions of this group and use of their valuable experiences and capabilities.
9. Specific attention to Non-communicable disease (NCDs) and chronic disease is another specification of THCP. Since the non-communicable diseases had the greatest burden in recent years (19), we created a surveillance and care system for selected chronic diseases (heart disease, chronic respiratory, depression and anxiety, diabetes, hypertension). Moreover, physiological risk factors such as blood sugar disorders, BMI, lipid profile and behavioral risk factors like nutrition, alcohol and smoking, physical activity and unsafe sex are monitored and controlled.
10. Empowerment of managers and staff of health complexes in relation to regional health management is very important strength of THCP. In this area, continuous training offered to providers about management of health data, monitoring, planning, quality improvement, epidemiology, research, and other required educations.

Besides the strengths, this new system has some limitations and weakness taken into consideration as follows: Resistance of some organizations and authorities in primary steps of project that needed to doubled efforts by research members and holding multiple meetings with all stakeholders (20).

## Conclusion

Health complex is effective and efficient model of public-private participation and practical solution to address many of the problems in the primary care system of the country. The project can organize the PHC system and family medicine program and realize the national vision of 20 years in the health sector (benefiting the health, welfare, food security, dignity and rights of human beings).

## Ethical considerations

Ethical issues (Including plagiarism, informed consent, misconduct, data fabrication and/or falsification, double publication and/or submission, redundancy, etc.) have been completely observed by the authors.
